# Similarity of Microplastic Characteristics between Amphibian Larvae and Their Aquatic Environment

**DOI:** 10.3390/ani14050717

**Published:** 2024-02-25

**Authors:** Michał Szkudlarek, Bartłomiej Najbar, Łukasz Jankowiak

**Affiliations:** 1 Department of Zoology, Institute of Biological Sciences, University of Zielona Góra, Profesora Zygmunta Szafrana 1, 65-516 Zielona Góra, Poland; 2 Doctoral School of Exact and Technical Sciences, University of Zielona Góra, Al. Wojska Polskiego 69, 65-762 Zielona Góra, Poland; 3 Department of Ecology and Anthropology, Institute of Biology, University of Szczecin, Wąska 13, 71-412 Szczecin, Poland

**Keywords:** sediment, water, polymer, color, microplastics, ecotoxicology, pollution, tadpoles

## Abstract

**Simple Summary:**

This study investigates the similarity of microplastic characteristics between amphibian larvae and their aquatic environment. Microplastics from water, sediment, and amphibian larvae were extracted and analyzed. This study focused on the shape, color, and chemical composition of the particles. The findings revealed a similarity in the microplastics’ shapes and colors between those in water and amphibian larvae and, to a lesser extent, between those in larvae and sediment. However, no significant similarities were observed in their chemical composition. This study highlights the impact of microplastic pollution on freshwater ecosystems and its implications for amphibian larvae.

**Abstract:**

Microplastics, pervasive environmental pollutants, are found across various ecosystems, including small inland water bodies. They are reported in different environmental media, yet little is known about the mutual relationships of microplastics’ properties across components of small inland water bodies. Here, having extracted and analyzed these particles from water, sediment, and amphibian larvae from 23 sites, we test within-site similarities regarding shape (morphological type), color, and chemical composition (polymer type). We also provide a brief characterization of the microplastics extracted from water and sediment regarding these parameters. We observed a statistically significant similarity of microplastics’ shapes and colors between those extracted from water and amphibian larvae. Such a similarity, though less pronounced, was also found between amphibian larvae and sediment. However, the chemical composition (polymer type) of the microplastics from water, sediment, and amphibian larvae did not exhibit any similarities beyond what would be expected by chance. The observed congruence in the colors and shapes of microplastics between amphibian larvae and their corresponding aquatic habitats underscores the profound interconnectedness among the constituents of freshwater ecosystems.

## 1. Introduction

Microplastics (MPs) are persistent and ubiquitous miniature particles of plastics. They often emerge from the fragmentation of plastic debris (secondary microplastics) [1]. As an emerging contaminant, microplastics have captured the attention of both the public and the scientific community. Originating from many sources, they can be found in a diverse range of shapes, colors, sizes, and chemical compositions (and thus densities), which all influence their distribution and fate in the environment. MP pollution, being a global problem, has been found in all compartments of the environment (air, rock, soil, water, sediment, and biota) and impacts both humans [2] and wild biota [3]. Interactions between organisms and microplastics can take various forms, such as ingestion [4,5], chemical leaching resulting from the “Trojan horse effect” [6], or adhesion [7]. The biological effects of MPs on individual organisms are very complex and multifaceted. They span a wide spectrum, affecting behavior [8], physiology [9,10,11], growth [12], morphology [13], histology [14,15], mortality [16], genotoxicity [17], and reproduction [18]. These effects are species- [14,19] and tissue-specific [20], being also modulated by accompanying stressors [21,22,23]. Moreover, they depend on the shapes of the microplastics, their sizes [14], and their chemical compositions [24]. The trophic transfer of these particles has been reported both in marine environments [25] and in an experimental setup encompassing larval *Ambystoma mexicanum* (Shaw & Nodder 1798) and zooplankton [26]. In brief, MPs affect many levels of biological organization.

The microplastic contamination of aquatic environments has mostly been studied in seas and oceans, with less focus on freshwater systems [27]. Furthermore, even though small inland water bodies have been found to be on average more heavily polluted with MPs than larger freshwater ecosystems [28], they have received comparatively less attention. Moreover, static and small inland water bodies, as opposed to open and dynamic ones, are more likely to accumulate microplastics rather than to serve as vessels of transportation into oceans [29]. With their crucial role in biodiversity and ecosystem services, coupled with an array of existential threats they face beyond pollution [30], freshwater ecosystems warrant closer scientific scrutiny. Previous research found the fiber and fragment shapes to be the most common for MPs in freshwater ecosystems, with polyethylene and polypropylene dominating among the polymer types [31,32].

The selectiveness in microplastics’ ingestion has been documented in fishes [33]. Theoretically, the properties of these particles in amphibian larvae should mirror the properties of MPs available to them (in water/sediment/food, as an encounter rate [34]) modulated by the interactions of specific feeding behaviors; larval sizes; and environmental conditions—e.g., availability of food [35]. Surprisingly though, the feeding ecologies of tadpoles across numerous species remain largely unexplored [36]. Nevertheless, based on the findings of Hu et al. [29], we expect that the characteristics of microplastics extracted from amphibian larvae will more closely resemble those found in water rather than those found in sediment. The shapes, colors, and chemical compositions of microplastics are of significance because they shed light on their origins, indicating potential sources, and thus proving valuable to nature conservation. Additionally, the similarities in MPs’ profiles across different species might provide supporting evidence for predation and the resulting trophic transfer of these particles. However, once ingested, the microplastics were documented to undergo changes like breaking down into smaller pieces [37]. Lastly, given that frogs have been experimentally observed to retain microplastics throughout metamorphosis [38], a comparative analysis of the MP characteristics between larvae, sediment, and water can aid in modeling microplastics’ transportation and fate.

To carry out such a comparative analysis, we first briefly characterized microplastics extracted from water and sediment. Then, we explored the potential mutual similarities concerning color, shape, and polymer type with those of the MPs extracted from amphibian larvae.

## 2. Materials and Methods

We chose amphibian larvae as our biotic study subjects due to their ecological sensitivity, crucial role in food webs, and their potential to serve as vectors of microplastics into terrestrial ecosystems [38].

Water, sediment, and dead amphibian larvae were collected from 23 sampling sites in lowland Western Poland (see the map in Figure S1; details in [39,40]). The sampling sites were diverse, consisting of desiccating inland water bodies such as ditches, ponds, and puddles (Figure S2). These sites varied from wild areas with minimal human impact to disturbed urban locations. Notably, some sites were heavily polluted with visible plastic debris. Prior to sample collection, all tools and containers (small stainless steel bucket, jars, and metal screw caps) were thoroughly rinsed with clean water and closed. Two liters of surface water was collected from each sampling site from a depth of 0–18 cm. This collection was accomplished using a small stainless steel bucket, which was submerged into the water to the specified depth. Upon retrieval, the water from the bucket was carefully poured into a pre-labeled, two-liter glass jar. Each jar was immediately sealed with a metal screw cap to prevent contamination and then stored at room temperature until the laboratory analysis took place. Secondly, using a stainless steel bucket, approximately one kilogram of wet bottom sediment was collected from each sampling site and transferred into a two-liter glass jar. Each jar was promptly sealed with a metal screw cap, labeled, and stored at −20 °C until the laboratory analysis took place. Finally, various amphibian larvae spanning ten taxa were collected from each site, totaling 914 individuals (for details, see [39,40]).

With the use of a water-jet pump, a vacuum flask, and a Büchner funnel, water from each sampling site was separately filtered through a 47 mm diameter, 20 μm porosity nylon filter (Merck Millipore NY2004700, Tullagreen, Carrigtwohill, Co., Cork, Ireland). The filtration continued until a significant decrease in flow was observed. In order to eliminate organic matter, the contents of each filter, presumed to contain both microplastics and organic matter, were flushed with 150 mL of analytically pure 30% (*v*/*v*) H_2_O_2_ into a separate 250 mL glass flask. This flask was then covered and placed in a CLW 15 STD (POL-EKO, Wodzisław Śląski, Poland) laboratory incubator, where it remained for a day at a temperature of 65 °C. Thereafter, the remaining liquid was stirred and filtered through a 47 mm diameter, 5 μm porosity polycarbonate filter (Whatman Nuclepore 10417412, Kent, UK). Each of these filters was then placed in its own individually labeled and covered glass Petri dish, where it remained until the time of analysis.

To extract potential microplastics from the sediments, we employed the methodology of Hu et al. [41] with modifications. First, the frozen jars containing sediments were thawed by removing them from the freezer and allowing them to sit at room temperature for a day. Following this, sediment from each jar was individually transferred to an aluminum pot and dried at a temperature of 65 °C inside a CLW 15 STD (POL-EKO, Wodzisław Śląski, Poland) laboratory incubator for a minimum period of one day. Subsequently, we proceeded with density separation using an analytically pure NaCl aqueous solution of 1.2 g/mL density. The dry sediment (30 g) from each site was separately mixed with 200 mL of the NaCl solution in a 250 mL glass beaker. This mixture was then stirred with a glass rod and left to sit overnight. The next day, the aqueous layer, approximately 125–150 mL, was decanted and filtered through a 20 μm porosity nylon filter (Merck Millipore NY2004700, Tullagreen, Carrigtwohill, Co., Cork, Ireland). The contents on this filter were then flushed with 150 mL of 30% (*v*/*v*) H_2_O_2_ into a glass flask. To aid in the digestion of organic matter, the flasks were covered and placed in a laboratory incubator set to 65 °C, where they remained for a day. Thereafter, the liquid content was stirred and filtered through a 47 mm diameter, 5 μm porosity polycarbonate filter (Whatman Nuclepore 10417412, Kent, UK). Each filter, holding the extracted particles, was then placed in an individually labeled and covered glass Petri dish for further analysis. After first decantation, each glass beaker was refilled with the NaCl solution up to 200 mL. The solution was then stirred with a glass rod and left overnight for a second extraction so as to ensure a comprehensive retrieval of all microplastics.

Methods of microplastic extraction from the amphibian larvae are detailed in our previous paper [39]. In short, the tadpoles were separately washed with clean water, cut into pieces, and digested with 30% (*v*/*v*) H_2_O_2_ in glass beakers. After incubation, the liquid content of the glass beakers was filtered onto 1.2 μm glass fiber filters (Whatman 1822-047, Kent, UK). Each filter was then carefully placed in its own lidded glass Petri dish.

The particle analysis was the same as the one detailed in our other papers [39,40]. We utilized a Nikon ECLIPSE Ni-U microscope (Tokyo, Japan), set to a magnification of 40×, equipped with a fiber optic illuminator, a Nikon DS-Fi2 camera (Tokyo, Japan), and NIS-Elements Br software (v. 4.30) to categorize each particle suspected of being a microplastic into one of the four discrete shape categories (morphological types): fiber, flake, granule, and fragment. Furthermore, we determined the dominant color of each potential microplastic, assigning it 1 of the 15 color categories [42]. To confirm the identity of the suspected microplastic particles, a subset of these particles was analyzed using a Nicolet^TM^ iS50 FTIR spectrometer equipped with an ATR module (Thermo Scientific, Waltham, MA, USA). For each measurement, we performed 50 scans within a 400–4000 cm^−1^ wavelength at a resolution of 4 cm^−1^. The resulting spectra were examined using OMNIC (v. 9.2.106) software, in conjunction with the Open Specy tool (v. 0.9.3) [43]. To confirm the identity of the putative microplastics, we matched their spectral profiles against a reference library of synthetic and semi-synthetic polymer spectra, requiring at least a 60% similarity for validation. In certain instances, we also carried out manual interpretation of spectra to verify the samples, as outlined by Hanke et al. [42]. This analysis enabled us to identify the chemical composition (polymer type) of the representative microplastic particles. Particles suspected to be microplastics, displaying notable similarities in appearance (such as color, size and shape) to the verified microplastics but which were not subjected to analysis, were also treated as microplastics.

The quality control measures undertaken to ensure the accuracy and reliability of results are the same as the ones detailed in one of our other papers [40].

Employing the R programming language (v. 4.1.3), we separately compared within-site microplastic color, shape (morphological type), and chemical composition (polymer type) matches in water, sediment, and amphibian larvae. Matches were counted when the same trait (e.g., blue color, the shape of a granule, or a polypropylene chemical composition) was found in both groups. As a control for the water–larvae and sediment–larvae analyses, we used matches with microplastics extracted from water and sediment from randomly selected sites (excluding self-comparisons), respectively. We utilized 999 permutations and averaged the number of matches. This number of permutations was chosen based on standard statistical practices to provide a balance between computational efficiency and reliability of results. To assess within-site similarities (average number of matches from comparisons) in the microplastic characteristics, we conducted a Chi-square goodness-of-fit test. This method enables testing similarities in the shape, color [44], and chemical composition of microplastics between groups. This test allows us to compare the observed average number of matches regarding MP characteristics within each site to the expected 1:1 ratio, under the null hypothesis that there are no within-site similarities.

## 3. Results

**Figure 1:**
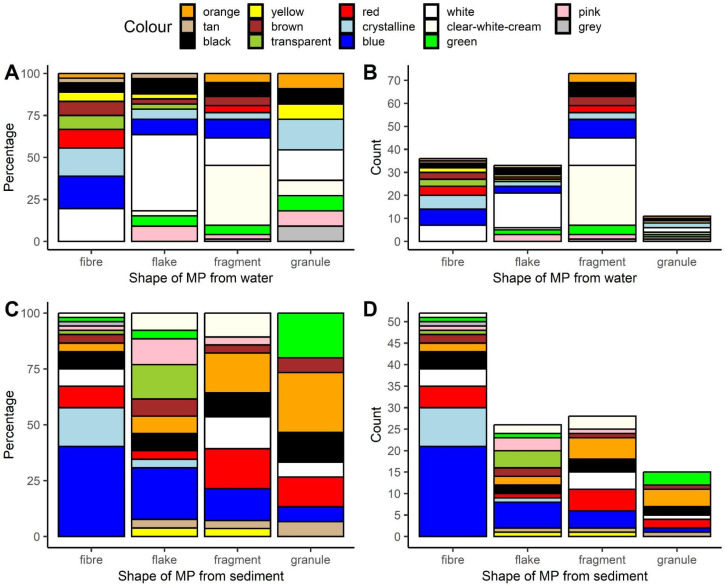
Color composition of differently shaped microplastics extracted from water (**A**,**B**), and sediment (**C**,**D**). Relative (**A**,**C**) and absolute abundance (**B**,**D**). This artwork was created with the ggplot2 package [45] in R.

We extracted 153 and 121 microplastics from water and sediment, respectively. The most common morphological type of those extracted from water was a fragment (approximately 48%, Figure 1B, Table S1), while for sediment, it was a fiber (approximately 43%, Figure 1D, Table S1).

The microplastics extracted from water and sediments were categorized into 14 out of the possible 15 color categories; the share of each is shown in Table S2, and their breakdown by specific shapes of MPs is illustrated in Figure 1A,C. In short, white was the most common color of the microplastics that were extracted from water (approximately 24%), while blue was the most common color for those extracted from sediment (approximately 26%).

**Figure 2:**
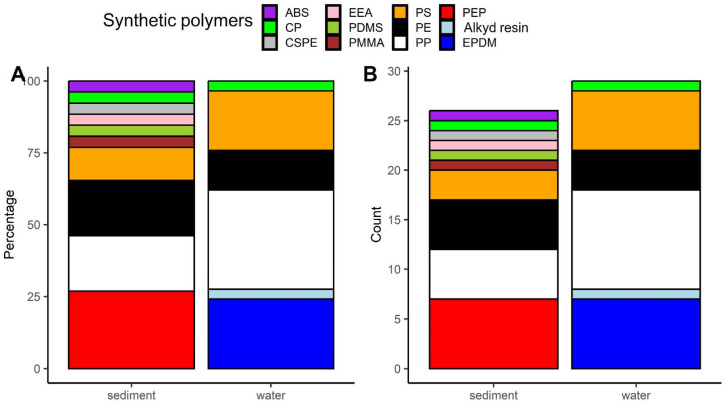
Share (**A**) and absolute abundance (**B**) of particular synthetic polymers detections among microplastics extracted from water and sediment. Abbreviations of synthetic polymers: ABS—acrylonitrile butadiene styrene; CP—cellophane; CSPE—chlorosulfonated polyethylene; EEA—ethylene ethyl acrylate; PDMS—polydimethylsiloxane; PMMA—poly(methyl methacrylate); PS—polystyrene; PE—polyethylene; PP—polypropylene; PEP—poly(ethylene:propylene); EPDM—poly(ethylene:propylene:diene) This artwork was created with the ggplot2 package [45] in R.

The spectroscopic analysis of the microplastics extracted from water and sediments revealed 12 synthetic polymers. Among the microplastics extracted from water, polypropylene was the most frequently identified one (approximately 34%, Figure 2A,B, Table S3), while for those extracted from sediment, poly(ethylene:propylene) was the most common polymer type (approximately 27%, Figure 2A,B, Table S3).

The respective findings for amphibian larvae are presented in one of our other papers [40]. In summary, among the microplastics extracted from tadpoles, the most frequently detected shape, color, and polymer type were fiber, blue, and polyethylene, respectively.

For colors, we observed a significant difference between the observed matches and control matches in both comparisons—between larvae and water, and between larvae and sediment. This indicates a similarity of microplastic color types in these respective pairings. The same significance effect and relation was found for shapes (Table 1). Interestingly, the similarity in both colors and shapes (number of matches) was less pronounced in the sediment–larvae comparisons. Regarding the chemical composition of microplastics (polymers), no significant difference was found between the observed matches and controls, as detailed in Table 1.

## 4. Discussion

We found fragments and fibers to be the most prevalent shapes of microplastics in water and sediment, respectively. This finding aligns with the results of Karaoğlu and Gül in a similar study [46]. However, it contrasts with the results of Hu et al. [28], who identified fibers and fragments as the predominant form of microplastics in water and sediment samples from small inland water bodies, respectively. This discrepancy could be attributed to the specific characteristics of the water bodies that we analyzed, some of which were visibly polluted with plastic debris. Such environments may harbor a higher proportion of fragments, predominantly secondary microplastics derived from larger plastic debris [47], as opposed to fibers, typically transported via stormwater runoff [48]. However, this hypothesis warrants further investigation.

Blue was the predominant microplastic color found in sediment, accounting for 26% of the analyzed particles, while in surface water, white microplastics dominated, representing 24% of the particles. Interestingly, blue microplastics also dominated among those extracted from amphibian larvae at these sampling sites [40]. However, white microplastics, despite their prevalence in surface water, were uncommon in the larvae [40]. This discrepancy raises questions about the putative avoidance of certain microplastics by amphibian larvae.

In our analysis, polypropylene emerged as the most commonly identified polymer (34%) among the microplastics extracted from the water samples that we tested. This finding is consistent with results from other parts of the world, where polypropylene was identified as one of the dominant polymers of waterborne microplastics (reviewed in [31,49]). Polypropylene, a widely used material in the production of various everyday items such as bottle caps, construction materials, automotive components, sports equipment, and packaging [50], often ends up constituting secondary microplastics due to improper disposal and subsequent fragmentation of plastic debris. In our study, poly(ethylene:propylene) was the most detected (27%) polymer of the microplastics extracted from sediments. Interestingly though, it was not detected by us among the microplastics extracted from surface water (Table S3).

Our study, which observed within-site similarities in the shapes and colors of microplastics extracted from amphibian larvae and water, partially corroborates the findings of Hu et al. [28], who reported a congruence in microplastics’ polymer and shape distributions between amphibian larvae and surrounding waters. This partial consistency in results could be explained by tadpoles’ intake of microplastics suspended in water rather than those in bottom sediment. Our observed lack of within-site concordance in the chemical composition of microplastics across water, sediment, and tadpoles calls for more in-depth research. It might be attributed to the fact that we sampled surface water, whose profile might overrepresent microplastics made of less dense polymers, as opposed to deeper water that amphibian larvae possibly ingest most microplastics from. It is crucial to model the distribution of microplastics, taking into account not only their chemical composition but also the shapes and sizes of the particles.

When interpreting the results of our study, we shall consider the possibility that certain shapes and sizes of microplastics may be less detectable in amphibian larvae because they are not bioaccumulated/retained. Instead, they are eliminated more readily than other shapes and size classes of MPs. The findings of Araújo et al. [51], who studied frog tadpoles, suggest that bigger microplastic particles accumulate in gills, medium-sized ones pass through the gastrointestinal tract and are egested from the cloaca, and the smallest microplastics seem to enter the bloodstream and accumulate within internal organs. Besides their size [52], the circularity of microplastic particles was also found to play a role in their translocation and accumulation [51]. In fishes, microplastic fibers were observed to have a higher retention time than other shapes [53]. This phenomenon is hypothesized to be responsible for the putative overrepresentation of this MP shape in studies on wild animals. Simultaneously, it suggests greater health risks posed by that particular shape of microplastic.

The scope of our study is limited by its regional and temporal focus of sampling, which may not fully reflect the broader patterns of microplastic pollution. Those are known to be shaped by, among others factors, annual dynamics of microplastic deposition rates into the sediments [54]. Methodological constraints, particularly in the size range of detectable microplastics, may have led to an underestimation of their presence. Furthermore, our analysis was restricted to amphibian larvae and did not include other species that might serve as bioindicators. These limitations highlight the need for expanded research to further elucidate the impacts of microplastics on freshwater ecosystems. Besides comparisons of MPs’ characteristics between sediment, water, and biota, future more complex approaches incorporating trophic and non-trophic species interactions [55] are needed. Lastly, environmental microplastic particles are known to harbor unique assemblages of bacteria and other microorganisms [56], which raises the question of preferential ingestion of such particles by tadpoles that are known to feed on microorganisms, an aspect that has not yet been studied.

## 5. Conclusions

Our study has provided important information about the properties of microplastics in various components of small inland water bodies. The observed significant similarities in the shapes and colors of MPs in water and amphibian larvae highlight the potential of these organisms as bioindicators for profiling microplastic pollution. Our work emphasizes the interconnectivity within these freshwater ecosystems and underscores the pivotal role that amphibians play in ecological surveillance. Moreover, this study can hopefully aid conservation, as the recognition of microplastic pollution profiles in water and the sediment of amphibian habitats is crucial for designing experimental exposure studies that mirror environmental conditions. Only such properly designed experiments will be able to shed light on the effects that this diverse class of pollutants has on animals.

For future research, we advocate a more nuanced approach that incorporates the feeding behaviors of a broader range of species. Moreover, the “Trojan horse effect” and the aspect of microplastics possibly serving as vectors of pathogens shall not be overlooked in future studies.

## Figures and Tables

**Table 1:** Results of Chi-square goodness-of-fit tests comparing number of observed matches (comparison of larvae with sample from water body) and control matches (comparison of larvae with a randomly selected water body sample, excluding self-comparisons) in two different matching settings (water-larvae and sediment-larvae). Color, shape, and polymers were analyzed. *p*-values below 0.05, indicating statistical significance, are in bold and marked with an asterisk.

		Observed	Control	χ^2^ Value	*p*-Value
color	water–larvae	803	658	14.391	<0.001 *
	sediment–larvae	807	707	6.605	0.010 *
shape	water–larvae	1563	1381	11.379	<0.001 *
	sediment–larvae	1438	1321	4.9616	0.025 *
polymers	water–larvae	62	72	0.74627	0.3877
	sediment–larvae	37	40	0.11688	0.7324

## Data Availability

The data presented in this study are available from the corresponding author on request. The data are not publicly available due to the ongoing analysis for future publication.

## Supplementary Materials

The following supporting information can be downloaded at https://www.mdpi.com/article/10.3390/ani14050717/s1, Figure S1: Location of the sampling sites. OpenStreetMap was used; Figure S2: Photographs of the main types of water bodies studied. A—puddle, B—ditch, C—pond; Table S1: Morphological type (shape) composition of the microplastics extracted from water and sediment. The values are rounded and given as % of the total; Table S2: Color composition of the microplastics extracted from water and sediment. The values are rounded and given as % of the total; Table S3: Chemical composition of microplastics extracted from water and sediment. The values are rounded and given as % of the total.
