# Supplementary material for: Similarity of Microplastic Characteristics between Amphibian Larvae and Their Aquatic Environment
Source: Animals (Basel). 2024 Feb 25;14(5):717. doi: 10.3390/ani14050717 (PMC10930510; doi:10.3390/ani14050717)
Supplement: Supplementary file 1 [file animals-14-00717-s001.zip › animals-2828113-supplementary.pdf]

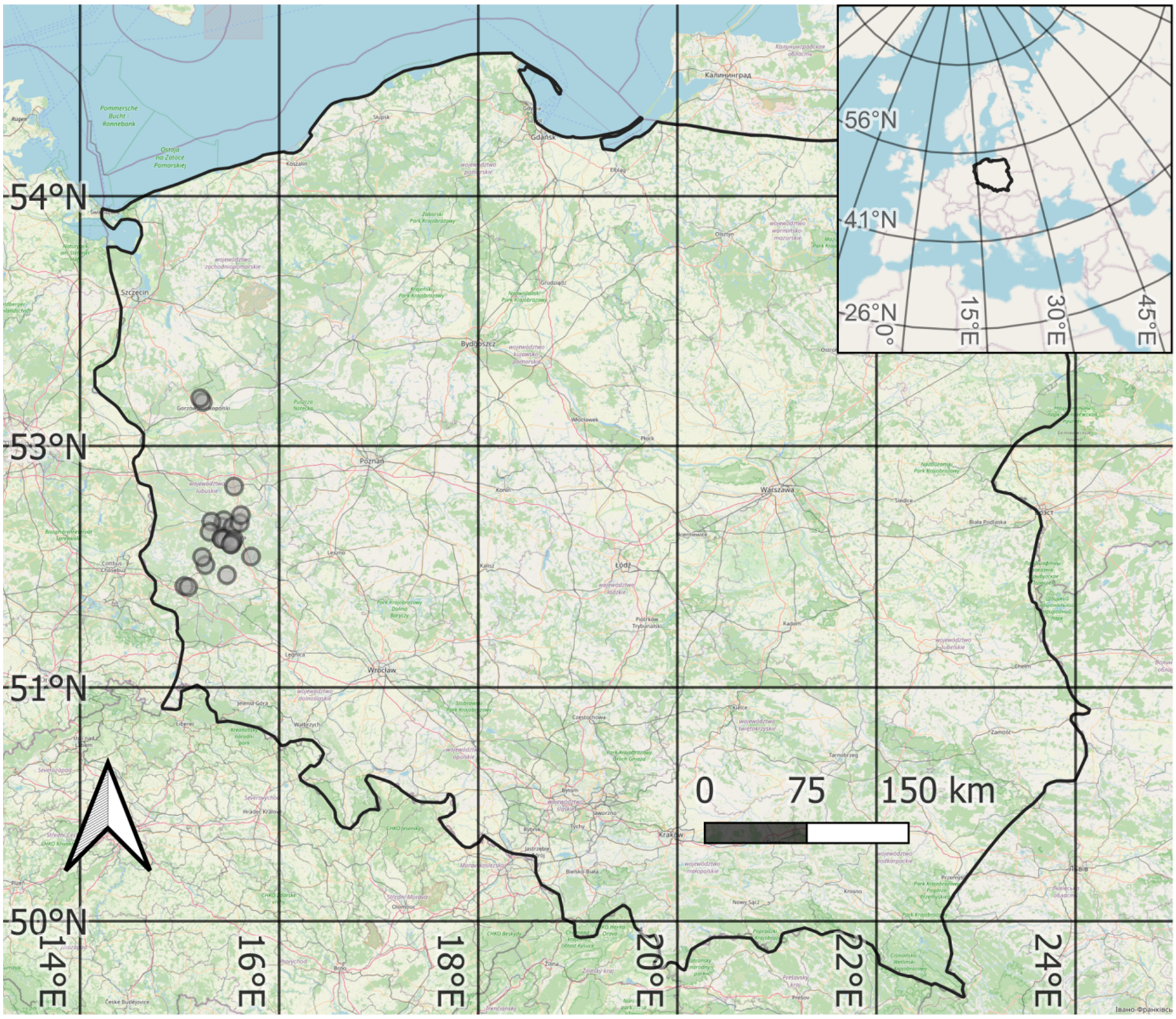

Figure S1. Location of the sampling sites. OpenStreetMap was used.

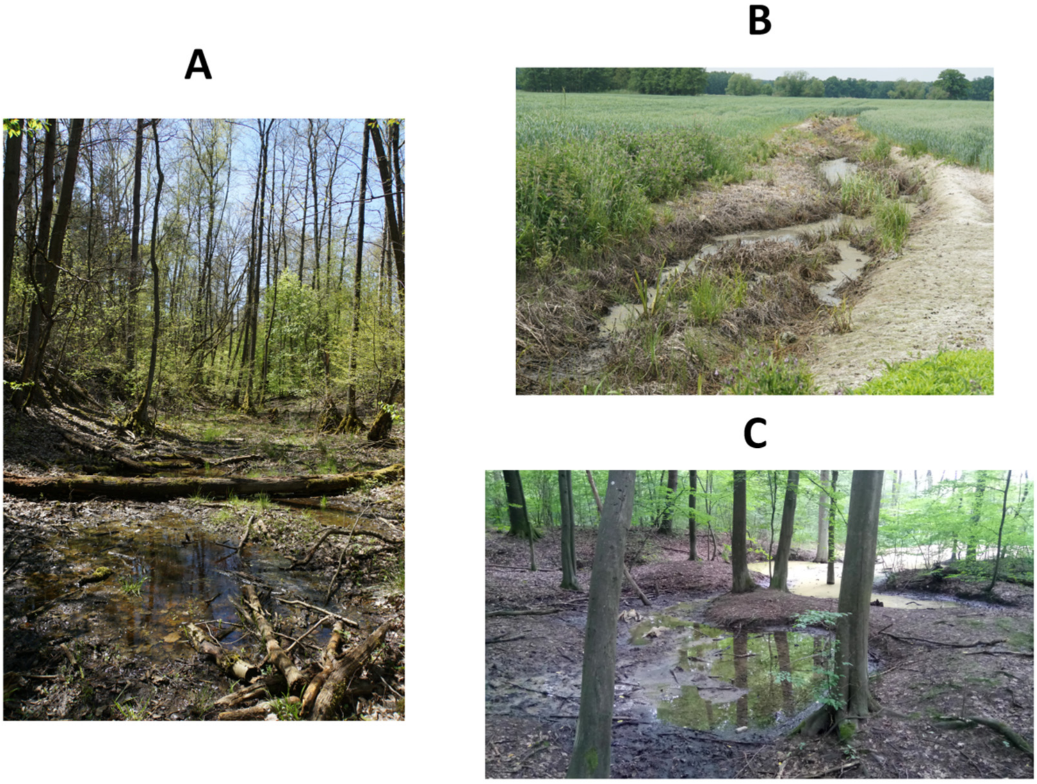

Figure S2. Photographs of the main types of water bodies studied. A – puddle, B – ditch, C – pond.

Table S1. Morphological type (shape) composition of the microplastics extracted from water and sediment. The values are rounded and given as % of the total.

	water	sediment
fragment	48	23
fiber	23	43
flake	22	22
granule	7	12

Table S2. Color composition of the microplastics extracted from water and sediment. The values are rounded and given as % of the total.

	water	sediment
blue	12	26
white	24	7
clear-white-cream	18	5
red	5	11
orange	4	11
black	8	9
crystalline	8	8
brown	5	5
green	5	4
pink	4	4
transparent	3	4
yellow	2	3
tan	1	2
grey	1	1

Table S3. Chemical composition of microplastics extracted from water and sediment. The values are rounded and given as % of the total.

	water	sediment
polypropylene	34	19
polyethylene	14	19
polystyrene	21	12
poly(ethylene:propylene)		27
poly(ethylene:propylene:diene)	24	
cellophane	4	4
acrylonitrile butadiene styrene		4
chlorosulfonated polyethylene		4
ethylene ethyl acrylate		4
polydimethylsiloxane		4
poly(methyl methacrylate)		3
alkyd resin	3	
